# How Much Is Too Much in a Reusable Laryngeal Mask Airway?

**DOI:** 10.7759/cureus.28921

**Published:** 2022-09-08

**Authors:** Abhishek Arya, Sonali Turki, Kamal Kajal

**Affiliations:** 1 Anaesthesiology, Pain Medicine and Critical Care, All India Institute of Medical Sciences, New Delhi, Delhi, IND; 2 Anaesthesia and Intensive Care, Postgraduate Institute of Medical Education and Research, Chandigrah, IND; 3 Anaesthesia and Intensive Care, Postgraduate Institute of Medical Education and Research, Chandigarh, IND

**Keywords:** n2o, nitrous oxide, airway, supraglottic airway, plma, proseal lma

## Abstract

ProSeal^TM^ Laryngeal Mask Airway (PLMA) (Teleflex Medical, Westmeath, Ireland) is a versatile and popular reusable supraglottic airway device. The manufacturer advises maximum reuse of up to 40 times. However, excessive reuse of the device can cause rare complications. Here, we report a rather uncommon complication of intraoperative cuff rupture of a PLMA probably due to the combination of a forceful metal introducer tip insertion and nitrous oxide-based anaesthesia.

## Introduction

ProSeal^TM^ Laryngeal Mask Airway (PLMA) (Teleflex Medical, Westmeath, Ireland) is a commonly used second-generation supraglottic airway device. Its salient features include an integrated bite block, a silicon peri-laryngeal cuff, an additional dorsal cuff, an integrated gastric drain tube, the availability of a metal introducer, and its reusability. Its reusability has made it a very popular airway device in resource-constrained settings.

However, the use of PLMA is also associated with complications. Common complications associated with PLMA include device insertion failure, gastric insufflation, malposition, dislodgement with loss of airway during the maintenance phase, regurgitation, and airway trauma [1]. Here, we share a rare event of intraoperative PLMA cuff rupture, its possible causes, and our troubleshooting experience.

## Case presentation

A 1-year-2-month-old patient (weight - 8 kg, height - 75 cm), with bilateral eye congenital cataract was posted for bilateral eye lens aspiration and posterior chamber intraocular lens implantation under general anaesthesia. After preanaesthetic checkup, the patient was transferred to the operating room. Inhalational induction was done using 50% Oxygen (O2) + 50% Nitrous-oxide (N2O) + 4% sevoflurane and intravenous access was obtained. After undergoing all the preliminary checks, a 1.5 size PLMA (used more than 40 times) was inserted orally using a metal introducer. The introducer was removed and the cuff was inflated by 6 mL air, however, the cuff pressure was not checked. The PLMA was connected to the ventilator. The Pressure Support mode of ventilation was selected with a pressure support of 10 cmH2O and a positive end expiratory pressure of 3 cmH2O. A minimal leak of 5 mL was noted. Anaesthesia was maintained with inhaled 50% O2 + 50% N2O + 2.5% sevoflurane. The surgery was initiated.

After 30 minutes of induction, a sudden loss of ventilation was noted. There was a sudden drop in delivered tidal volume with a loss of capnography trace. Inspired O2 was increased to 100%. Ventilator circuitry and all the connections were checked. A negative check enforced emergency undraping of the child to resecure the airway. The PLMA was removed and the patient was mask ventilated with 100% O2. There was no desaturation or hemodynamic instability during this time. Upon removal, the PLMA cuff was found to be ruptured and it was replaced by a 1.5 size Ambu® Aura 40^TM^ reusable LMA (Anbu A/S, Ballerup, Denmark). Mechanical ventilation was restored. Further surgery proceeded uneventfully. The child was awakened at the end of the surgery, LMA was removed and the child was monitored in the post-anaesthesia care unit (PACU). No adverse events were noted and the child was discharged from PACU in an active, healthy state.

**Figure 1:**
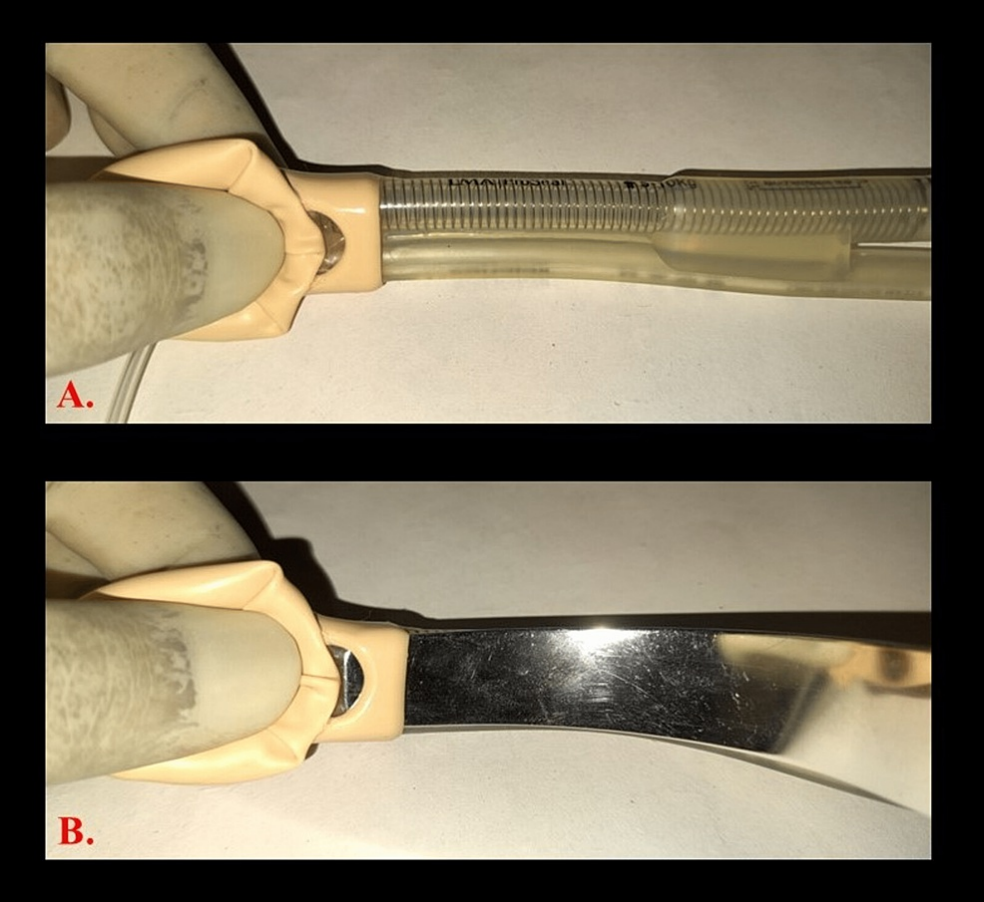
Base of the cuff of an unruptured PLMA. A. PLMA with an unruptured cuff without metal introducer in situ. B. PLMA with an unruptured cuff with a metal introducer in situ. PMLA: ProSeal^TM^ Laryngeal Mask Airway

**Figure 2:**
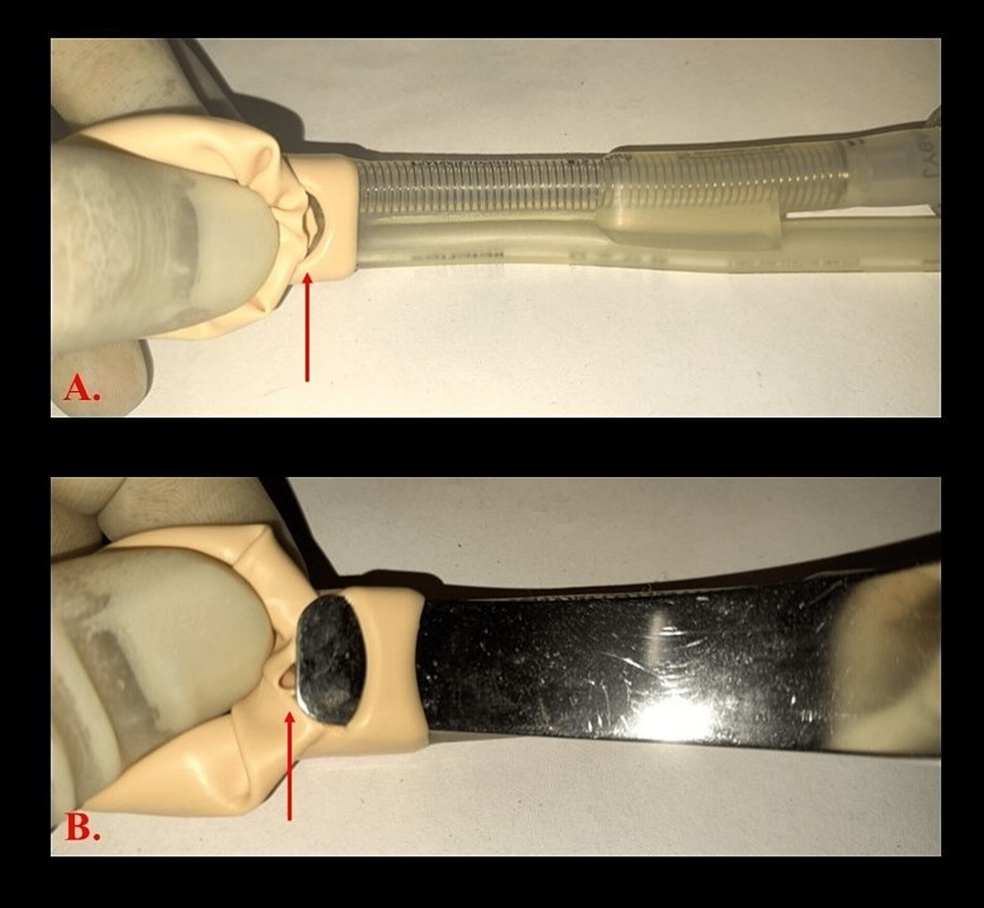
Base of the cuff of the ruptured PLMA. A. PLMA with ruptured cuff without metal introducer in situ. B. PLMA with ruptured cuff with metal introducer in situ. (Arrows mark the rupture site.) PMLA: ProSeal^TM^ Laryngeal Mask Airway

The PLMA was re-examined and compared to another PLMA after the case (Figures 1, 2). On examining the cuff of the PLMA, the rupture site was found at the base of the cuff in the introducer strap corresponding to the site where the metal introducer tip is placed.

## Discussion

PLMA is a reusable device. The manufacturer advises maximum reuse of up to 40 times [2]. Though a documented safe reuse of the device has greatly exceeded this number, its excessive reuse adds to rare complications [3]. Maniar et al. reported a case of fracture of PLMA after excessive use [4]. Doneley et al. also showed that the lifespan of the PLMA is shorter than the Classic^TM^ LMA (Teleflex Medical, Westmeath, Ireland) [5].

The use of nitrous oxide while administering anaesthesia has also been associated with an increase in the cuff pressure of PLMA [6,7]. Sharma et al. attempted to solve this problem. In their study, they found that PLMA cuff inflation with 50% O2 and 50% nitrous oxide mixture provided more stable cuff pressures [8].

In our case, the intraoperative cuff rupture was hypothesized to be associated with three primary factors. Firstly, the position of the cuff rupture corresponded to the site where the tip of the metal introducer is placed. A forceful impaction of the metal introducer tip could have weakened the silicone cuff at this site, though it was not apparent during the pre-introduction routine checks. Secondly, the cuff was ruptured 30 minutes into the procedure while using 50% O2 + 50% N2O inhaled anaesthesia. Nitrous oxide administration during anaesthesia is known to increase cuff pressures in a PLMA as discussed above and it has a probable association with the intraoperative cuff rupture at an already weakened site in an overused PLMA as in our case. Thirdly, the PLMA was reused more than the manufacture's recommendation of 40 times.

## Conclusions

PLMA is a versatile and popular reusable supraglottic airway device. However, excessive reuse of the device can cause rare complications. Our report shares a rather uncommon complication of intraoperative cuff rupture of a PLMA possibly due to the combination of a forceful metal introducer tip insertion and nitrous oxide usage for administering anaesthesia. We draw multiple lessons from our experience. Firstly, the manufacturer advises two primary methods of PLMA insertion with either a metal introducer or finger manipulation. Our experience suggests that finger manipulation may be a better method to insert a PLMA that has been reused many times. Secondly, rechecking cuff pressures in nitrous oxide-based anaesthesia is advisable. Lastly, excessive reuse of equipment is a rampant practice in many resource-constrained settings, however, it can pose significant patient safety issues. Therefore, a balance is needed to prevent jeopardizing patient safety.
